# The PROVENT-C19 registry: A study protocol for international multicenter SIAARTI registry on the use of prone positioning in mechanically ventilated patients with COVID-19 ARDS

**DOI:** 10.1371/journal.pone.0276261

**Published:** 2022-12-30

**Authors:** Silvia De Rosa, Nicolò Sella, Emanuele Rezoagli, Giulia Lorenzoni, Dario Gregori, Giacomo Bellani, Giuseppe Foti, Tommaso Pettenuzzo, Fabio Baratto, Giorgio Fullin, Francesco Papaccio, Mario Peta, Daniele Poole, Fabio Toffoletto, Salvatore Maurizio Maggiore, Paolo Navalesi, The SIAARTI Study Group

**Affiliations:** 1 Department of Anesthesiology and Intensive Care, San Bortolo Hospital, Vicenza, Italy; 2 Department of Medicine, Anesthesia and Critical Care Unit, Padua University Hospital, Padua, Italy; 3 Department of Medicine and Surgery, University of Milano-Bicocca, San Gerardo Hospital, Monza, Italy; 4 Unit of Biostatistics, Epidemiology and Public Health, Department of Cardiac, Thoracic, Vascular Sciences and Public Health, Padua University School of Medicine, Padua, Italy; 5 Anaesthesia and Intensive Care Unit, Ospedali Riuniti Padova Sud "Madre Teresa Di Calcutta" Hub Covid Hospital Monselice (Padova)-ULSS 6 Euganea, Padua, Italy; 6 Anesthesia and Critical Care Unit, Ospedale dell’Angelo, Mestre, Italy; 7 Anesthesia and Critical Care Unit, Ospedale Ca’ Foncello, Treviso, Italy; 8 Anesthesia and Critical Care Unit, Ospedale di Belluno, Belluno, Italy; 9 Anaesthesia and Intensive Care Unit, Ospedali di San Donà di Piave e Jesolo, San Donà di Piave, Italy; 10 Department of Innovative Technologies in Medicine & Dentistry, Section of Anesthesia and Intensive Care, G. D’Annunzio University, SS. Annunziata Hospital, Chieti, Italy; Hualien Tzu Chi Hospital, TAIWAN

## Abstract

**Background:**

The worldwide use of prone position (PP) for invasively ventilated patients with COVID-19 is progressively increasing from the first pandemic wave in everyday clinical practice. Among the suggested treatments for the management of ARDS patients, PP was recommended in the Surviving Sepsis Campaign COVID-19 guidelines as an adjuvant therapy for improving ventilation. In patients with severe classical ARDS, some authors reported that early application of prolonged PP sessions significantly decreases 28-day and 90-day mortality.

**Methods and analysis:**

Since January 2021, the COVID19 Veneto ICU Network research group has developed and implemented nationally and internationally the “PROVENT-C19 Registry”, endorsed by the Italian Society of Anesthesia Analgesia Resuscitation and Intensive Care…’(SIAARTI). The PROVENT-C19 Registry wishes to describe 1. The real clinical practice on the use of PP in COVID-19 patients during the pandemic at a National and International level; and 2. Potential baseline and clinical characteristics that identify subpopulations of invasively ventilated patients with COVID-19 that may improve daily from PP therapy. This web-based registry will provide relevant information on how the database research tools may improve our daily clinical practice.

**Conclusions:**

This multicenter, prospective registry is the first to identify and characterize the role of PP on clinical outcome in COVID-19 patients. In recent years, data emerging from large registries have been increasingly used to provide real-world evidence on the effectiveness, quality, and safety of a clinical intervention. Indeed observation-based registries could be effective tools aimed at identifying specific clusters of patients within a large study population with widely heterogeneous clinical characteristics.

**Trial registration:**

The registry was registered (ClinicalTrial.Gov Trials Register NCT04905875) on May 28,2021.

## Introduction

### Background and rationale

Severe acute respiratory syndrome coronavirus 2 (SARS-CoV-2) infection is characterized mainly by moderate/severe pneumonia associated with progressive endothelial damage and coagulopathy [1]. Acute respiratory failure occurs in 42% of patients with Coronavirus disease 2019 (COVID-19) pneumonia, and among them 61–81% of patients tense to intensive care unit (ICU) admission [2].

Prone position (PP) is a well established treatment for acute respiratory distress syndrome (ARDS), that has been use for many years and is now recommended for invasively ventilated patients with moderate to severe ARDS [3]. Indeed, PP proved to have several beneficial effects on effects on patients with ARDS. Firstly, the recruitment of the dorsal lung region improves tidal ventilation and increases end-expiratory lung volume and chest wall elastance [4]. Second, PP promote a more homogeneous distribution of lung stress and strain, with less over-distension in non-dependent lung regions and less cyclical opening and closing in dependent lung regions, that allows lung protective ventilation and avoids ventilation induce lung injury [5, 6]. Finally, PP improves ventilation-perfusion match, decreasing alveolar shunt [7]. Since the preliminary studies on PP in patients with ARDS, PP has consistently demonstrated to lead to a significant improvement in oxygenation that is sustained even when supine position is re-established [8]. Later, the PROSEVA trial [9], trial [9] across 466 patients with ARDS found found that 28-day mortality was 16% in the prone group and 33% in the supine group (p < 0.001), with an hazard ratio for death with prone position of 0.39 (95% confidence interval 0.25–0.63). More recently, a meta-analysis on eight randomized trials with a total of 2129 patients showed use of PP was only in moderate to severe ARDS and conducted for greater than 12 h per day was associated with a mortality benefit with a relative risk of 0.74 (95% confidence interval 0.56–0.99).

Despite these promising evidence and the guidelines recommendation [10], the use of PP was low in the real-world clinical practice until COVID-19 pandemic [11]. Recently, despite the lack of clinical trials or cohort studies in a large population, prone position (PP) was recommended as an adjuvant therapy for improving ventilation in patients with COVID-19 ARDS by the Surviving Sepsis Campaign COVID-19 guidelines [12–14].

In patients with COVID-19 requiring mechanical ventilation, the PRONA-COVID group reported that PP was widely used in this population and that overall PP led to a significant increase in oxygenation, although no significant change in respiratory mechanics was reported [15]. In a recent prospective cohort study performed in awake, non-intubated patients with COVID- 19-related pneumonia and requiring oxygen supplementation, PP was feasible and effective in rapidly ameliorating blood oxygenation. The effect was maintained after resupination in half of the patients [16]. However, the nursing workload and the adverse events of PP, such as pressure ulcers, facial edema, endotracheal tube obstruction/ accidental removal, neuropathies, compression of nerves and retinal vessels, should also be considered [17]. Furthermore, limited information is available on the role of the duration of PP and whether prolonged PP might improve gas exchange, respiratory mechanics and outcomes of patients with COVID-19 [18, 19].

### Objective

Since January 2021, the COVID19 Veneto ICU Network research group has developed, implemented, and nationally and internationally spread the “PROVENT-C19 Registry”, endorsed by the Italian Society of Anesthesia Analgesia Resuscitation and Intensive Care (SIAARTI) with the aim to describe the subpopulations of invasively ventilated patients with COVID-19 that benefit the most from PP therapy (ClinicalTrials.gov Identifier: NCT04905875). This web-based registry will provide a clear example of translational medicine and translational research where data from clinical practice feed a database for clinical research and, at the same time, the database research tools may improve daily practice.

## Methods

### Study design

Since January 2021, the SIAARTI Society has developed, implemented, nationally and internationally promoted the “PROVENT-C19 Registry”, endorsed by Coordination of Intensive Care - Veneto Region. The Ethics Committee of the St. Bortolo Hospital, Vicenza, Italy (Study ID Numbers: 22/21) and all other participating centers have approved the protocol. Each eligible participant has provided written informed consent. For each enrolled patient, their personal information were been recorded and kept in a secure folder. To protect their privacy, the patients’ personal information were only made accessible to the researchers. The registry was registered (ClinicalTrial.Gov Trials Register NCT04905875) on May 28,2021; the study was designed in accordance with the Declaration of Helsinki. The PROVENT registry with protocol amendment has gone international and has obtained company endorsements European Society of Intensive Care Medicine (ESICM) and European Society of Anaesthesiology and Intensive Care (ESAIC). Almost fifty centers joined the registry and a number of registered patients that has exceeded a thousand for which the results will certainly be encouraging.

### Eligibility criteria

This registry enrolls all patients diagnosed with COVID-19 infection requiring invasive mechanical ventilation and undergoing PP who meet the inclusion (but not the exclusion) criteria. Each hospital will designate two intensivists for patient enrollment. Inclusion criteria are the following: Adult patients (age > 18 years old) fulfilling all the following inclusion criteria can be included in this study: Laboratory-confirmed COVID-19 infection; Pronation of intensive care patients undergoing invasive mechanical ventilation. Exclusion criteria were contraindications to PP and patients in PP but undergoing non-invasive ventilation must be excluded. Please note that the lack of consensus regarding the timing and duration of PP leads to variability in clinical practice, and treatment is undertaken depending on the decision of the clinician in charge. In these circumstances, it is advisable to keep the inclusion criteria as broad as possible to obtain an accurate picture of clinical practice worldwide.

(Table 1). Informed consent is not an eligibility criterion because data will be processed in a pseudonymised manner in accordance with European Union General Data Protection Regulation (EU-GDPR).

**Table 1:** Inclusion and exclusion criteria.

Inclusion Criteria	Exclusion Criteria
◼ Age ≥18 years ◼ A laboratory-confirmed COVID-19 infection using Reverse Transcription Polymerase Chain Reaction ◼ Admission to ICU for invasive Mechanical Ventilation ◼ A clinical indication for PP	◼ Contraindications to prone positioning

### Outcomes

The Primary Outcome is represented by the identification of patient profiles in terms of outcomes based on specific PP models and their intermediate surrogate endpoints. However, Secondary Outcomes are:

- Gas exchange and respiratory mechanics before and after the first positioning manoeuvre and during the initial and final phases of PP. A positive response was defined a priori as an increase in partial oxygen tension (PaO2)/ fractional inspired oxgen (FiO2) ≥20%
- Pao2 / Fio2, before and after each PP manoeuvre. A positive response was defined a priori as an increase in the Pao2 / Fio2 ratio of ≥20%
- Duration of PP
- Ventilatory parameters (tidal volume, ventilatory frequency, Positive End-Expiratory Pressure, plateau pressure and static compliance of the respiratory system) after repeated prone positioning
- Mortality: ICU mortality and hospital mortality, 28 days of free ventilation
- All clinical variables at each PP cycle (starting with the patient’s Case Report Forms)
- The clinical variables relevant for the application of PP treatment will be described, as well as the starting time of the treatment. In particular, the absolute and relative frequencies of those clinical variables relevant to the application and the starting time of PP treatment will be described
- PP usage rates (number of PP admissions / ICU) will be described in terms of annual absolute frequencies and cumulative incidence among all patients enrolled from all participating centres

### Timeline

**Fig 1:**
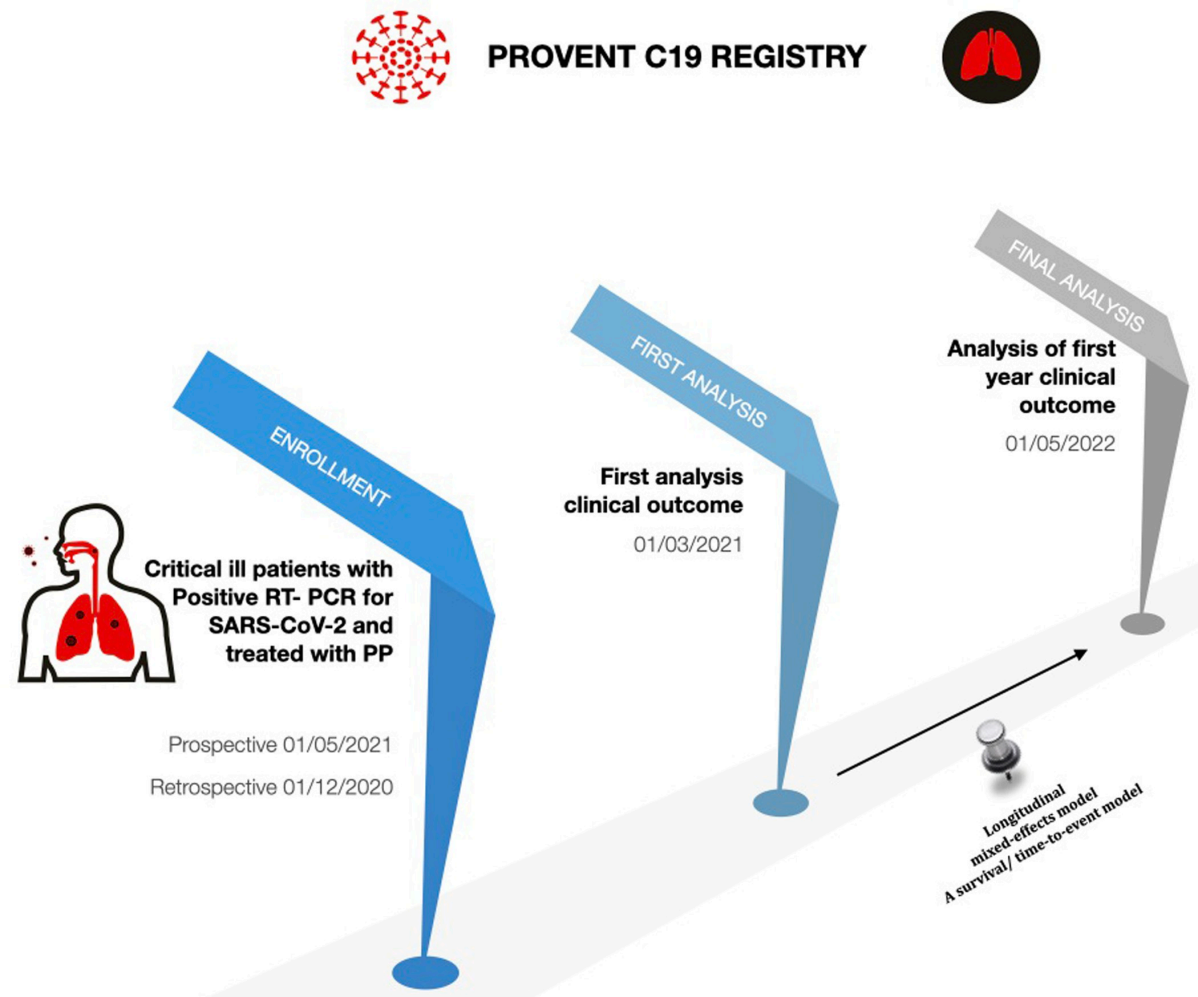
Timeline of the study.

The study recruits all patients diagnosed with COVID-19 infection requiring invasive mechanical ventilation and undergoing PP at the participating centers will be retrospectively or prospectively enrolled. Patient enrollment will coincide with PP treatment start date (independently prescribed by the attending physician according to widely accepted guidelines and local clinical practice). Study duration is one year, starting presumably from 01/03/2021. Patients enrollment at each center will start at different times depending on the spread of the registry and local IRB approval. The first patient might be expected to be enrolled prospectively on 01/05/2021. Retrospectively patient might be enrolled since 01/12/2020. Final results of this study will be available in 31/12/2022. Fig 1 shows the flowchart for the study timeline. Patients will be monitored and data recorded until hospital discharge. Post-discharge follow-up has not been envisaged.

### Statistical analysis

Descriptive statistics will be reported using I quartile/median/III quartile for continuous variables and percentages (absolute numbers) for categorical variables. Wilcoxon-Kruskal-Wallis and Chi-squared tests will be employed to compare the distribution of continuous and categorical variables, respectively.

Joint models will be estimated to evaluate the association between PP maneuvers and short and long -term outcomes. Traditionally, linear mixed models for longitudinal data, i.e., changes in respiratory parameters collected repeatedly during the ICU stay according to clinical choice, and Cox Proportional Hazard models for time-to-event outcomes, i.e., in-hospital death, are estimated separately. However, such an approach does not allow for taking into account of the dependency between respiratory parameters changes and the outcome of interests. Joint models overcome such a limitation since they allow for modelling longitudinal and time-to-event data simultaneously [20]. The basic structure of a joint model consists of two components, the longitudinal and the time-to-event ones. Specifically, in the present study, the model will include the estimation of:

◆ A longitudinal mixed-effects model that analyses changes in the respiratory parameters collected repeatedly during the ICU stay [21];
◆ A survival or time-to-event model which analyses the time until the in-hospital death [22, 23].

The joint estimation of these sub-models will be performed by assuming a mutual correlation between repeated observations through individual-level random-effects parameters. All these analyses might guide indications for PP in patients with COVID-19 to personalize treatments and to improve patients’ long-term outcomes.

The workflow methodology and software solution of research electronic data capture (REDCap) [21, 22] was used to construct the PROVENT-C19 Registry. REDCap (Research Electronic Data Capture) is a secure, web-based software platform designed to support data capture for research studies, providing 1) an intuitive interface for validated data capture; 2) audit trails for tracking data manipulation and export procedures; 3) automated export procedures for seamless data downloads to common statistical packages; and 4) procedures for data integration and interoperability with external sources. Clinical data from patients with COVID-19 registered to the ICU of participant centers will be included the registry. These data will be converted to the electronic case report form (eCRF) format used in REDCap. Primary data collection based on source documents will be performed clearly and accurately by site personnel trained on the protocol and eCRF completion. eCRF data will be collected for all patients enrolled. Data validation, quality control, and database management will be conducted to ensure data integrity. For the project duration, the principal investigator will maintain complete and accurate documentation including but not limited to the following: clinical, instrumental, biochemical, genetic and genomic data, and any other information required to substantiate data entered into the CRF, project progress records, laboratory reports, electronic case report forms. Analyses will be performed using R software [24].

### Data collection

The following data will be collected anonymously on a case report form: Date and time of admission; Demographic data and comorbidities; Type of respiratory support in progress before intubation; Clinical parameters and respiratory exchanges before intubation and analytical blood gas parameters; assessment of the performance of several organ systems in the body (Sequential Organ Failure Assessment Score); Consciousness assessment (Glasgow Coma Score); Evaluation of blood chemistry tests (platelets count, bilirubin, serum creatinine, potassium, sodium, amount of urine);Total number of pronation and for each pronation cycle will be assessed arterial blood gas values at T1 (Supine arterial blood gases pre-pronation), T2 (First Arterial blood gases in PP), T3 (Last arterial blood gases in PP), T4 (First arterial blood gases in Supine Positioning Post-Pronation), T5 (Last arterial blood gases in Supine Positioning Post-Pronation). Long-term outcome after discharge from intensive care and complications (Pressure ulcers, Endotracheal tube obstruction or accidental removal, Barotrauma) related to PP will be recorded. Local investigators will transfer all the data to an electronic collection card that coincides with the paper card (eCRF, Research Electronic Data Capture—RedCap). Each local investigator received instructions on how to use the eCRF and a personal username and password. Each patient will be identified through a patient identification number (PIN).

## Utility and discussion

**Fig 2:**
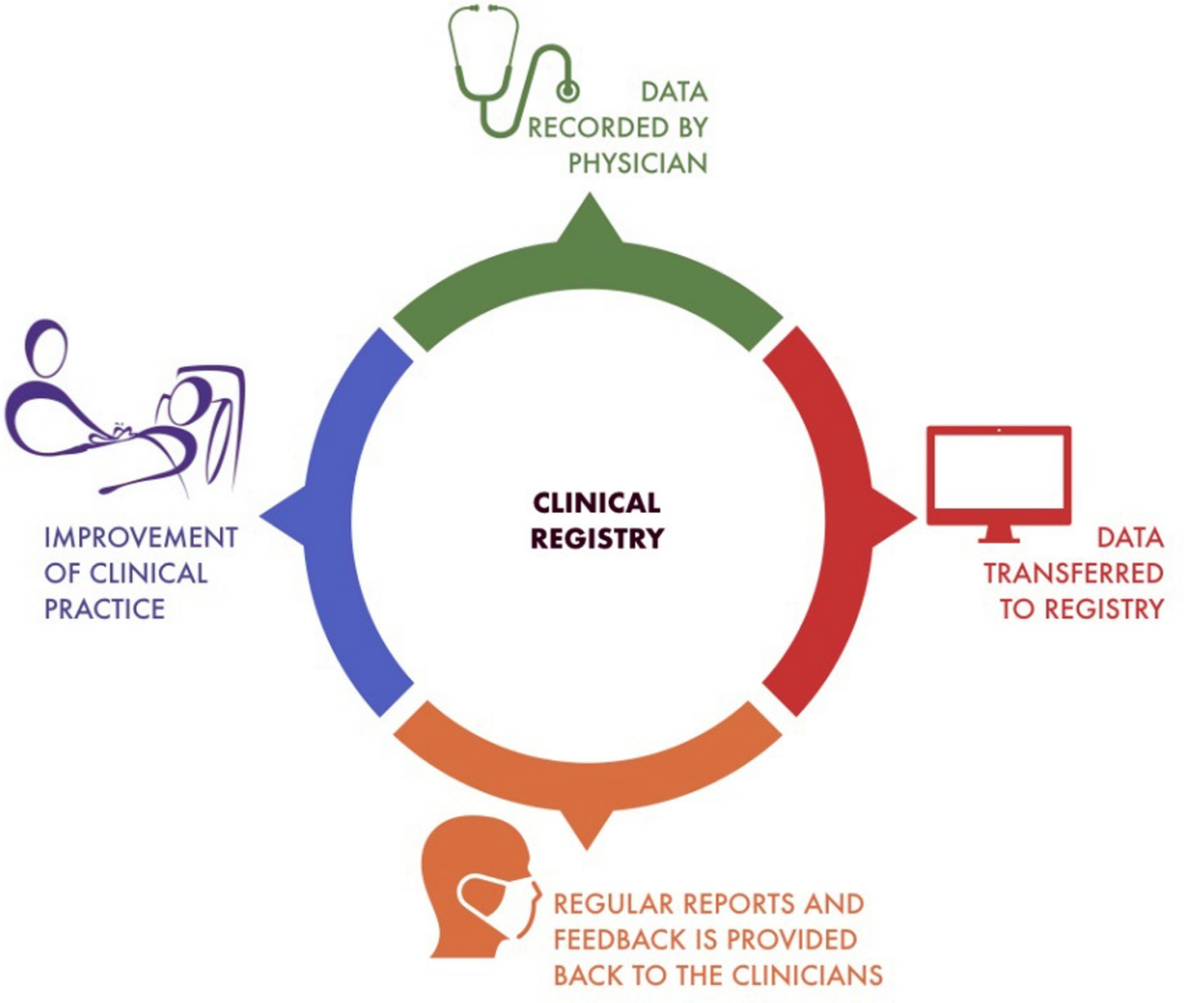
Clinical registry.

The term "registry" emphasizes and enhances the aspect of data retention with the aim of describing epidemiological relationships and differences, supporting quality assurance and improvement, as well as clinical research. In addition, registry should allow an evaluation of efficacy in medical clinical practice, but also a monitoring of patient safety as well as economic evaluation [Fig 2]. The web platform used for the registry—designed specifically for research purposes and powered by prospective observed clinical data—promotes actively a good clinical practice with an active interaction between physicians and researchers. The purpose of the registry is also to obtain a specific report on the specific clinical practice of each center also for the purpose of "support" in making clinical decisions. Observational studies have several limitations not allowing a comprehensive assessment of real clinical practice (i.e. restricted inclusion and exclusion criteria, complexity of health care settings, and variability in study population characteristics or inferential methods). Ethical issues and difficulties related to data variability can be overcome through utilization of a web-based platform. Observation-based registries have limited costs and effective tools able to identify specific clusters of patients within a large study population with widely heterogeneous clinical characteristics.

Clinical quality records enable to monitor the quality of health care provided to the present specific group of patients with COVID-19 by collecting, analysing and reporting relevant health information, but also identifying benchmarks for clinical performance and related variation in clinical outcomes. Generally, ongoing reporting of clinical data from the registry completes the clinical outcome feedback loop which is a defining feature of clinical quality registries. Feedbacks have been identified as one of the most effective interventions to improve practices and management in health care institutions. Clinical feedbacks provided by the web-based platform of this international registry will represent a summary of a care performance over a given period of time that can be transmitted a posteriori to the healthcare professional in any form. These feedbacks, by objectifying the level of individual and collective performance, will encourage a potential modification in practice improving performance. The efficacy of usual treatments performed in the ICU (such as mechanical ventilation) is easily perceived by the physician and local coordinators. On the other hand, for treatments with restricted indications (as PP in patients with COVID-19), clinical efficacy is based on the results of few PP performed over a broad period of time. An objective recording process of specific outcomes and patients/treatments characteristics allow the physician to go over a subjective perception of efficacy. Stratification for other confounding factors recorded in this registry (e.g., disease aetiology and/or clinical scoring system for organ dysfunction) may further increase the reliability of the overall treatment effectiveness. A clinical quality registry could be an opportunity to improve understanding of pathophysiologic mechanisms and to facilitate both predictive and prognostic enrichment [25, 26].

## Conclusions

The rationale behind the PROVENT-C19 Registry recognizes a new approach based on the real-life observation of the application of the PP for invasively ventilated patients with COVID-19. This approach is based on the concept of precision medicine based on the implementation of a large multidisciplinary and multi-parametric database aimed at identifying clusters of patients with COVID-19 with specific features who would benefit the most from PP, based also on real-time clinical feedbacks as clinical decision support system useful for improving process of care or patient outcomes.

### Registry status

The Registry l started in October 2021 in the coordinating centre, the St. Bortolo Hospital, Vicenza, Italy with the inclusion of the first patient. Fifty-nine other centres in Italy joined the registry. As of June 2021, the amendment was approved as an international registry. Several other centres have indicated that they are interested in participating (ricerca@siaarti.it; https://www.siaarti.it).

## Supporting information

S1 File (PDF)

S2 File (PDF)

S1 Checklist SPIRIT 2013 checklist: Recommended items to address in a clinical trial protocol and related documents*. (DOC)

## List of abbreviations

ARDS: Acute Respiratory Distress Syndrome
COVID-19: Coronavirus disease 2019
eCRF: electronic Case Report Form
PP: prone positioning
SIAARTI: Italian Society of Anesthesia Analgesia Resuscitation and Intensive Care
