# Supplementary material for: The PROVENT-C19 registry: A study protocol for international multicenter SIAARTI registry on the use of prone positioning in mechanically ventilated patients with COVID-19 ARDS
Source: PLoS One. 2022 Dec 30;17(12):e0276261. doi: 10.1371/journal.pone.0276261 (PMC9803226; doi:10.1371/journal.pone.0276261)
Supplement: S1 File — (PDF) [file pone.0276261.s001.pdf]

OBSERVATIONAL STUDY PROTOCOL

Title: Prone Positioning for invasively ventilated patients with COVID-19: an interactive, web-based, multicenter, observational registry

Protocol Acronym: PRone pOsitioning for inVasively vENTilated patients with COVID-19 Registry (PROVENT-C19 Registry)
Protocol version: 01
Protocol version date: 17/03/2021

Proponent: The study is proposed by the Italian Society of Anesthesiology, Analgesia, Resuscitation and Intensive Care (SIAARTI) and is endorsed by Coordination of Intensive Care - Veneto Region.

Coordinating Site: Anesthesia and Critical Care Unit, San Bortolo Hospital, Vicenza, Italy

STUDY STAFF

Coordinating Investigator: Silvia De Rosa[1]

Data Management: Dario Gregori[2], Giulia Lorenzoni[3]

Steering Committee: Coordination of Intensive Care - Veneto Region (Paolo Navalesi[4], Giorgio Fullin[5], Dario Gregori[6], Mario Peta[7], Daniele Poole[8], Fabio Toffoletto[9]), Giacomo Bellani[10], Giuseppe Foti[11], Francesco Papaccio[12], Tommaso Pettenuzzo[13], Emanuele Rezoagli[14], Nicolò Sella[15]

Participating Centers

The dissemination phase constitutes an integral part of the study and thus the final number of centers involved cannot be predicted a priori. Key professionals have been contacted who will be responsible for the spread and implementation of this registry-based study in their respective center. The Institutional Review Board (IRB) will be regularly updated by the Sponsor-Investigator as new centers join the study.

---

[1] Anesthesia and Critical Care Unit, San Bortolo Hospital, Vicenza, Italy
[2] Unit of Biostatistics, Epidemiology and Public Health, Department of Cardiac, Thoracic, Vascular Sciences and Public Health, Padua University School of Medicine, Italy.
[3] Unit of Biostatistics, Epidemiology and Public Health, Department of Cardiac, Thoracic, Vascular Sciences and Public Health, Padua University School of Medicine, Italy.
[4] Department of Medicine, Anesthesia and Critical Care Unit, Padua University Hospital, Padua, Italy
[5] Anesthesia and Critical Care Unit, Ospedale dell'Angelo, Mestre, Italy
[6] Unit of Biostatistics, Epidemiology and Public Health, Department of Cardiac, Thoracic, Vascular Sciences and Public Health, Padua University School of Medicine, Italy.
[7] Anesthesia and Critical Care Unit, Ospedale Ca' Foncello, Treviso, Italy.
[8] Anesthesia and Critical Care Unit, Ospedale di Belluno, Belluno, Italy
[9] Anaesthesia and Intensive Care Unit, Ospedali di San Donà di Piave e Jesolo, San Donà di Piave, Italy
[10] Department of Medicine and Surgery, University of Milano-Bicocca, San Gerardo Hospital, Monza, Italy
[11] Department of Medicine and Surgery, University of Milano-Bicocca, San Gerardo Hospital, Monza, Italy
[12] Anesthesia and Critical Care Unit, Ospedale dell'Angelo, Mestre, Italy.
[13] Department of Medicine, Anesthesia and Critical Care Unit, Padua University Hospital, Padua, Italy
[14] Department of Medicine and Surgery, University of Milano-Bicocca, San Gerardo Hospital, Monza, Italy
[15] Department of Medicine, Anesthesia and Critical Care Unit, Padua University Hospital, Padua, Italy

Table of Contents

Background e rationale 4

Objective 6

Study design 6

Study population 7

Study outcomes 8

Data management 9

Subsequent use of data 11

Statistical Analysis Plan 11

Safety and adverse events 11

Administrative/Regulatory aspects 12

Ethical aspects 12

Publication and dissemination policy 14

References 15

Background e rationale

SARS-CoV-2 infection is characterized mainly by moderate/severe pneumonia associated with progressive endothelial damage and coagulopathy. Acute respiratory failure among COVID-19 occurs in 42% of patients with COVID-19 pneumonia, and 61–81% of patients requiring intensive care [1]. Among the suggested treatments for the management of ARDS patients, prone position (PP) can be used as an adjuvant therapy for improving ventilation in these patients, as recommended in the Surviving Sepsis Campaign COVID-19 guidelines [2-4]. The main rationale of PP is the recruitment of dorsal lung regions, increasing end-expiratory lung volume and chest wall elastane, decreasing alveolar shunt, and improving tidal ventilation [5]. In patients with severe ARDS, early application of prolonged PP sessions significantly decreased 28-day and 90-day mortality. The reduced mortality, however, is not only determined by an increase in arterial oxygen tension, but also by the benficial effect of PP to reduce the damage associated with mechanical ventilation (VILI) [6-8].

PP can reduce mortality only if prescribed in case of severe impaired oxygenation, early, for prolonged periods, and associated to a protective ventilation strategy, as shown by the following meta-analyses. Munshi et al. [9] in a meta-analysis, evaluated the effect of prone positioning on 28-day mortality compared with conventional mechanical ventilation in supine position in adults with ARDS. The meta-analysis revealed no difference in mortality. However, subgroup analyses reported that PP can lead to a drop in the rate of mortality among patients with severe ARDS when applied to patients for least 12 hours a day. Mora-Arteaga et al. [10] In their meta-analysis, showed that PP can only reduce mortality in ARDS in the presence of: 1. low tidal volume ventilation; 2. PP onset within the first 48 hours; and 3. severe hypoxemia.

In a recent prospective cohort study performed in awake, non-intubated patients with COVID-19-related pneumonia and requiring oxygen supplementation [11], PP was feasible and effective in rapidly ameliorating blood oxygenation. The effect was maintained after resupination in half of the patients. Therefore, a careful patient selection, an adequate clinical protocol and pronation technique are essential to provide an effective treatment. Furthermore, prolonged pronation beyond the standard 16-18 hours has been proposed. Among those patients with longer prone ventilations (median time 36 hours), oxygenation seem to improve steadily [12]. The study was pivotal, carried on ten patients, and thus could only generate an

hypothesis, which is however clinically meaningful, identifying a novel approach that is starting to spread in critical care.

However, the adverse events of PP and the nursing workload should also be considered. Adverse effects associated with PP are several such as pressure ulcers (second-degree pressure sores or higher), bruising around the mouth due to presence of the tracheal tube, edema around the eyes and facial edema, endotracheal tube obstruction or accidental removal, limited access to the venous route, displacement of indwelling catheter and chest tube, gastroesophageal reflux, hyper-salivation, neuropathies, compression of nerves and retinal vessels [13].

However, although only prospective observational studies can be accurate to assess the effects of PP on a determined clinical outcome, several limitations do not allow a comprehensive assessment of real clinical practice (i.e. restricted inclusion and exclusion criteria, complexity of health care settings, and variability in study population characteristics or inferential methods). Ethical issues and difficulties related to data variability can be overcome through utilization of a web-based platform: observation-based registries are inexpensive and effective tools able to identify specific clusters of patients within a large study population with widely heterogeneous clinical characteristics. Since January 2021, our research group (Coordination of Intensive Care - Veneto Region) has developed, implemented, and nationally spread the "PROVENT-C19 Registry" with the aim to describe the subpopulations of invasively ventilated patients with COVID-19 that benefit the most from PP therapy. This web-based registry will provide a clear example of translational medicine and translational research where data from clinical practice feed a database for clinical research and, at the same time, the database research tools may improve our daily clinical practice.

Recently, the COVID-19 pandemic has been characterized by high prevalence of patients with severe multiorgan dysfunction, high mortality rate, lack of ICU resources, and the need for ICU discharge in a rapid, but safe, manner. PP have been proposed as an adjuvant therapy for improving ventilation in these patients (the Surviving Sepsis Campaign COVID-19 guidelines [4]). Nevertheless, no data is currently available on application and feasibility of PP in invasively ventilated patients with COVID-19 on their outcomes.

Objective

Primary Objective

The primary objective of this study is to define potential clusters of critically ill patients – treated with PP – that are homogeneous in terms of both clinical and treatment characteristics.

Secondary Objectives

Secondary objectives of this study are:

1) To assess the association between cluster membership and positive short-term outcome (i.e. an improvement in respiratory parameters)
2) To assess the association between cluster membership and positive long-term outcomes, defined here as 28days ventilator free days and patient survival at ICU discharge
3) To describe oxygenation, assessed by PaO_2/FiO_2 ratio, before and after each PP manoeuvre
4) To describe the clinical circumstances under which clinicians opt for PP
5) To describe PP utilization rates in intensive care units in Italy
6) To describe PP in terms of treatment duration in intensive care units in Italy
7) To describe PP in terms of treatment timing in intensive care units in Italy
8) To investigate the impact of time of prone position ventilation on PaO_2/FiO_2 ratios after each PP cycle and on days free form ventilation
9) To investigate the impact of PP on renal outcome

Study design

This is an national, multicenter, non-profit, retrospective and prospective observational cohort study. The study is non interventional. Study duration is one year, starting presumably from 01/03/2021. Patients enrollment at each center will start at different times depending on the spread of the registry and local IRB approval. The first patient might be expected to be enrolled prospectively on 01/05/2021. Retrospectively patient might be enrolled since 01/12/2020. Final results of this study will be available in 01/05/2022.

Setting

All patients undergoing PP at the participating centers will be retrospectively or prospectively enrolled. Patient enrollment will coincide with PP treatment start date (independently prescribed by the attending physician according to widely accepted guidelines and local clinical practice). Patients will be monitored and data recorded until hospital discharge. Post-discharge follow-up has not been envisaged.

This is an observational study: enrolled patients will not receive additional treatments other than those routinely administered in ICUs. Nonetheless, all parameters related to PP treatment will be recorded in the web-based registry and analyzed a posteriori to reveal potential correlations with patient outcomes.

Deviations from the study plan

Any deviations from the present protocol will be reported to the local Institutional Review Board by the Principal Investigator through submission of an amendment request to be reviewed by IRB according to local/internal regulations.

Early termination and interruption of clinical evaluation

Beside reporting the start and end dates of the study for monitoring purposes, the Principal Investigator will inform the IRB of the early termination of the study and inherent reasons.

Study population

Inclusion criteria

Adult patients (age >18 years) who meet all the following inclusion criteria may be included in this study:

1. Laboratory-confirmed COVID-19 infection
2. Prone positioning in patients admitted to ICU undergoing invasive Mechanical Ventilation

It should be underlined that the lack of consensus concerning the timing and duration of PP leads to variability in clinical practice and treatments are initiated in accordance with the judgement of the responsible physician. Under these circumstances, it is preferable to keep

inclusion criteria as wide as possible so as to obtain a real picture of the clinical practice worldwide.

Exclusion criteria

Bedsides contraindications to the Prone Positioning and patients in PP but undergoing Non-invasive ventilation should be excluded.

Patient Withdrawal from Study

Any patient may withdraw from this study at any time without prejudice to their care by directly informing the physician in charge of the registry. Patients do not have to disclose their reasons for withdrawal. Being an observational study, in case of patient withdrawal from study, his/her clinical pathway will remain unchanged.

Study outcomes

Primary Endpoint

To identify profiles of patients in terms of outcomes according to specific patterns of PP and their intermediate surrogate endpoints.

Secondary Endpoint

Secondary endpoints of the study are:

1. Gas exchanges and respiratory mechanics before and after the first positioning manoeuvre, and during the early and late phase of the prone positioning. A positive response was defined a priori as an increase in $Pao_2/Fio_2 \geq 20\%$
2. Pao_2/Fio_2, before and after each prone positioning manoeuvre. A positive response was defined a priori as an increase in Pao_2/Fio_2Pao 2/Fio 2 ratio ≥20%.
3. Duration of PP
4. ventilatory parameters (tidal volume, ventilatory frequency, PEEP, plateau pressure and static compliance of the respiratoty system) after repeated prone positioning.
5. Mortality: ICU Mortality and Hospital mortality, 28-day free ventilator days
6. All Clinical variables at each PP cycle (as from the patient CRFs).
7. Clinical variables relevant to the application of PP treatment will be described, as well as timing of initiation of treatment. Specifically, absolute and relative frequencies of those clinical variables relevant to the application and timing of initiation of PP treatment will be described.

8. PP utilization rates (number of PP/ICU admissions) will be described in terms of yearly absolute frequencies and cumulative incidence among all the enrolled patients from all participating centers*.*

Sample size

Since this is an observational registry, no definite preliminary sample size calculation would be required. Considering the anticipated participation of 100 centers, an estimation of 1000 included patients seems reasonable.

Enrollment procedure

Adult patients of both sexes admitted to ICUs with COVID-19 admission diagnosis and who are in invasive mechanical ventilation with prone position will be considered eligible for enrollment.

Follow-up procedure

Clinical variables will be assessed and recorded during hospitalization only. Post-discharge follow-up has not been envisaged in this study.

End of participation in the study

At Hospital discharge, data collection and thus participation in the study will end for each respective patient. The scheduled end of the study is defined as the date on which the last enrolled patient is discharged, according to study duration.

Data management

Data property

The individual data provided by a participating ICU are primarily the property of the ICU who generated the data. All investigators have the right to access their data at any time.

Data collection

Data collection serves a scientific purpose. The data will be generated in the participating centers and recorded via a web application on the servers of the University of Padova using the study management software RedCap®.

Study data will be collected and managed using REDCap [15,16] electronic data capture tools, developed, tested, validated, and hosted at the Department of Cardiac-Thoracic-Vascular Sciences and Public Health, University of Padova, Italy. REDCap (Research Electronic Data Capture) is a secure, web-based software platform designed to support data capture for research studies, providing 1) an intuitive interface for validated data capture; 2) audit trails for tracking data manipulation and export procedures; 3) automated export procedures for seamless data downloads to common statistical packages; 4) procedures for data integration and interoperability with external sources.

Data will be recorded in the e-CRF only using coding procedures described in a separate document to comply with data protection applicable laws, including EU GDPR. A coding key reporting patient's name, surname, and date of birth will be filed only in local site files together with a subject screening and enrollment log.

In cases where a medical institution is interested in participation, an account in the RedCap platform is created. The data will be recorded using an encrypted data connection (HTTPS) in input masks via a web browser or mobile app. To ensure pseudonymized data analysis, each patient will be assigned a unique Subject ID (Patient Identification Number). RedCap is a secure web application for building and managing online databases. By using a hierarchical, role-based access concept, unauthorized access to the data will be impossible. Access to RedCap will be granted only to data collecting staff of participating centers, in accordance with the procedures outlined in the present protocol. These persons are bound to secrecy.

Data management and retention

For data management, the study management software RedCap will be used. Name-related identification of individual patients by the documentation center (e.g., the University of Florence) is not required at any time during the study. During data collection and data processing, therefore, all necessary measures will be taken in order to establish de facto anonymization. The University of Padova will also be responsible for data storage. The data storage facilities are located in a locked, central room, accessible only to system administrators. Each participating center is granted access only to the patient data it has generated and recorded on the RedCap platform. Each participating center is expected to provide periodic ad hoc reports on the data it has collected.

Subsequent use of data

SIAARTI and the steering committee, on behalf of the investigators, have the right to use all data that are pooled in the databank for scientific purposes. Investigators will be regularly informed about ongoing study activities. The steering committee and the SIAARTI Study Group have the right to access the data, pooled in the databank, for research purposes after the research project has been terminated, and with the approval of the SIAARTI Scientific Committee.

Archiving

A copy of the electronic databank will be kept in Department of Cardiac-Thoracic-Vascular Sciences and Public Health, University of Padova, Italy and preserved for 5 years for subsequent use by SIAARTI and Steering Committee.

Statistical Analysis Plan

All eligible patients enrolled in the registry will be included in the analyses. Continuous variables will be reported as median and Interquartile Range (IQR). Categorical variables will be reported as percentages. Statistical tests may be carried out for exploratory purposes, as appropriate.

Joint models will be used [14] to estimate the association of pronation with short- and long-term outcomes. Joint models consist of the simultaneous estimation of two statistical models that traditionally would have been estimated separately. The model will include the estimation of:

1) A longitudinal mixed-effects model that analyses changes in the respiratory parameters collected repeatedly during the ICU stay;

2) A survival or time-to-event model which analyses the time until the in-hospital death.

The joint estimation of these sub-models will be performed by assuming a mutual correlation between repeated observations through individual-level random-effects parameters.

Safety and adverse events

This observational study will be conducted according to the current protocol. This is a non interventional observational study. Advice on treatment is not provided by this registry-based

study. Complications and adverse events that occurred while delivering PP treatment will be also recorded in the registry.

Administrative/Regulatory aspects

Economic aspects:

No additional costs will be charged to the Health System.

Insurance

Given the observational nature of the study and that there is no deviation from the standard of care in each participating center, there is no need for an additional insurance policy to that already provided for normal clinical practice.

Ethical aspects

This observational study will be conducted and reported according to the current protocol, in agreement with the Dir. EU 2001/20/EC relating to the implementation of good clinical practice in the conduct of clinical trials on medicinal products for human use, the principles published in the *Good Clinical Practice* (GCP) guidelines, and the ethical principles set out in the World Medical Association Declaration of Helsinki as a recognized ethical basis.

The Investigator ensures that the patients' personal data will be protected and processed in accordance with The General Data Protection Regulation (EU) 2016/679 (GDPR) on data protection and privacy.

Informed Consent and Data Processing

This observational study involves critically ill patients who frequently lack capacity to provide informed consent for processing his/her personal data. Considering that:

- The study is intended to promote the health of the group represented by the study population
- Because of the expected high severity of illness in the study population, it has to be assumed that most patients will have diminished capacity to understand all information and provide the informed consent

- The study objectives cannot be accomplished using data from individuals who have the full capacity to provide the informed consent or using other research methodologies

In cases where oral or written informed consent prior to data collection cannot be obtained, the Informed Consent will be obtained from the patient's guardian or legally authorized surrogate in accordance with art. 6 of the Italian Decree n. 101/2018 for the national implementation of General Data Protection Regulation (EU) 2016/679. The presumed will of the patient should be considered. The patient's consent is recorded in the patient file. Patients or their legal representatives have the right to withdraw their consent and to interrupt participation in the study at any time and without giving reasons. All patients who regain their ability to consent for participation in the study should provide their informed consent to continue in the study, as long as their health condition during hospitalization permits. If a formerly incapacitated patient decides at this time or later not to participate in the study, he/she may withdraw his/her from participation at any time and without stating a reason, without incurring any disadvantages for his/her further medical treatment.

The participating centers have to observe their locally established way of proceeding for including patients incapable of giving informed consent and follow the recommendations of the local IRB.

Data collection occurs upon early stage pseudonymization of the patients considered eligible for participation in this study. Participating centers draw up local patient identification lists and allocate a unique multidigit number to each patient. Only pseudonymized patient-related medical data are transferred from the participating center to the documentation center; no information that allows identification of patients is revealed to the documentation center. To ensure the principle of data minimization, processing of personal data will be adequate, relevant and limited to what is necessary in relation to the purposes of this study (art. 5, par. 1, lett. c of EU Regulation 2016/679).

Institutional Review Board and Competent Authorities

The Investigator will provide the local Institutional Review Board and Competent Authorities with the study protocol and any relevant document related to patient participation in this study (Informative Note and Informed Consent). Any activity correlated to the study may only be started if the Institutional Review Board and Competent Authorities have approved it. The Principal Investigator will be notified, in writing, of the IRB's decision. Any modifications to the approved protocol will be reported to the local IRB by the Investigator through submission of an amendment request to be reviewed by IRB according to local/internal regulations.

Publication and dissemination policy

Role and responsibilities of the Principal Investigator and Co-Investigators

The Principal Investigator has drafted the study protocol. Co-Investigators and their collaborators (if any) will be responsible for collecting patients data at the participating centers. The Principal Investigator will be responsible for managing, analyzing, and interpreting the data.

Clinical data from all the centers involved will be analyzed every 6 months and presented in abstracts at international conferences or published as preliminary reports on COVID-19 patients.

Intellectual Property Right

Being an independent study, the Sponsor-Investigator owns the intellectual property rights in it, in agreement with the Italian Ministerial Decree 17 December 2004, Art. 1, comma 2, letter c.

Publication policy

The Principal Investigator is responsible for the conduct of the study and data processing. In agreement with ICH-GCP, the Principal Investigator agrees to produce a yearly report on the study and publish all data generated from this study irrespective of results. The Principal Investigator will ensure that data are properly reported, and research findings are disseminated responsibly. Data dissemination and communication through scientific publications and/or presentations at congresses and conferences, as well as participation in multicenter studies, will follow statistical analysis of anonymized data. Publication of the data

takes place in an aggregated form only. Information about individual patients will not be published or shared. Results will be presented in abstract at international conferences. After project completion, a series of peer-review publications will be produced to present cumulative data on patient outcomes and clinical variation during PP treatment, and to describe clusters of patients that benefit the most from PP treatment. The Steering Committee (Paolo Navalesi, Silvia De Rosa, Giacomo Bellani, Giuseppe Foti, Giorgio Fullin, Dario Gregori, Giulia Lorenzoni, Francesco Papaccio, Tommaso Pettenuzzo, Daniele Poole, Emanuele Rezoagli, Nicolò Sella, Fabio Toffoletto) will provide:

- ✓ contribution to the conception and design of the registry;
- ✓ coordination and supervision for the accurancy of the inserted data and the compliance to the ethical reccomendations;
- ✓ responsability for all aspects of the work to ensure that issues related to the accuracy or integrity of part of the work are adequately investigated and answered.

A group authorship ("SIAARTI Study Group") will be created, including all the investigators of the participating centers. The number of investigators for each center will be related to the number of the included patients and to the quality of data collection. Every five patients with complete records, one investigator will be assured to the respective center.

References

1.Acar, H.C., Can, G., Karaali, R. et al. An easy-to-use nomogram for predicting in-hospital mortality risk in COVID-19: a retrospective cohort study in a university hospital. BMC Infect Dis 21, 148 (2021).

2.Yang X, Yu Y, Xu J, et al. Clinical course and outcomes of critically ill patients with SARS-CoV-2 pneumonia in Wuhan, China: a singlecentered, retrospective, observational study. Lancet Respir Med 2020; 8: 475–81.

3.Bouadma L, Lescure FX, Lucet JC, Yazdanpanah Y, Timsit JF. Severe SARS-CoV-2 infections: practical considerations and management strategy for intensivists. Intensive Care Med 2020; 46: 579–82.

4. Alhazzani W, Møller MH, Arabi YM, et al. Surviving Sepsis Campaign: guidelines on the management of critically ill adults with coronavirus disease 2019 (COVID-19). Intensive Care Med 2020; 46: 854–87.
5. Kallet RH. A Comprehensive Review of Prone Position in ARDS. Respir Care. 2015 Nov;60(11):1660-87.
6. Broccard AF, Shapiro RS, Schmitz LL, Ravenscraft SA, Marini JJ. Influence of prone position on the extent and distribution of lung injury in a high tidal volume oleic acid model of acute respiratory distress syndrome. Crit Care Med 1997; 25: 16–27.
7. Valenza F, Guglielmi M, Maffioletti M, et al. Prone position delays the progression of ventilator-induced lung injury in rats: does lung strain distribution play a role? Crit Care Med 2005; 33: 361–67.
8. Cornejo RA, Díaz JC, Tobar EA, et al. Effects of prone positioning on lung protection in patients with acute respiratory distress syndrome. Am J Respir Crit Care Med 2013; 188: 440–48.
9. Munshi L, Del Sorbo L, Adhikari NKJ, Hodgson CL, Wunsch H, Meade MO, Uleryk E, Mancebo J, Pesenti A, Ranieri VM, Fan E. Prone Position for Acute Respiratory Distress Syndrome. A Systematic Review and Meta-Analysis. Ann Am Thorac Soc. 2017 Oct;14(Supplement_4):S280-S288.
10. Mora-Arteaga JA, Bernal-Ramírez OJ, Rodríguez SJ. The effects of prone position ventilation in patients with acute respiratory distress syndrome. A systematic review and metaanalysis. Med Intensiva. 2015 Aug-Sep;39(6):359-72.
11. Coppo A, Bellani G, Winterton D, Di Pierro M, Soria A, Faverio P, Cairo M, Mori S, Messinesi G, Contro E, Bonfanti P, Benini A, Valsecchi MG, Antolini L, Foti G. Feasibility and physiological effects of prone positioning in non-intubated patients with acute respiratory failure due to COVID-19 (PRON-COVID): a prospective cohort study. Lancet Respir Med. 2020 Aug;8(8):765-774.
12. Carsetti, A., Damia Paciarini, A., Marini, B. et al. Prolonged prone position ventilation for SARS-CoV-2 patients is feasible and effective. Crit Care 24, 225 (2020).
13. Lucchini A, Bambi S, Mattiussi E, Elli S, Villa L, Bondi H, Rona R, Fumagalli R, Foti G. Prone Position in Acute Respiratory Distress Syndrome Patients: A Retrospective Analysis of Complications. Dimens Crit Care Nurs. 2020 Jan/Feb;39(1):39-46.
14. Wulfsohn MS, Tsiatis AA. A joint model for survival and longitudinal data measured with error. Biometrics. 1997;330–9.

15. Harris PA, Taylor R, Minor BL, Elliott V, Fernandez M, O'Neal L, et al. The REDCap consortium: Building an international community of software platform partners. J Biomed Inform. 2019 Jul;95:103208.
16. Harris PA, Taylor R, Thielke R, Payne J, Gonzalez N, Conde JG. Research Electronic Data Capture (REDCap) - A metadata-driven methodology and workflow process for providing translational research informatics support. J Biomed Inform. 2009 Apr;42(2):377–81.
