# Supplementary material for: The PROVENT-C19 registry: A study protocol for international multicenter SIAARTI registry on the use of prone positioning in mechanically ventilated patients with COVID-19 ARDS
Source: PLoS One. 2022 Dec 30;17(12):e0276261. doi: 10.1371/journal.pone.0276261 (PMC9803226; doi:10.1371/journal.pone.0276261)
Supplement: S2 File — (PDF) [file pone.0276261.s002.pdf]

REGIONE DEL VENETO
ULSS8
BERICA

Servizio Sanitario Nazionale - Regione Veneto
AZIENDA ULSS N. 8 BERICA
Viale F. Rodolfi n. 37 – 36100 VICENZA
COD. REGIONE 050–COD. U.L.SS.508 COD.FISC. E P.IVA 02441500242–Cod. iPA AUV
Tel. 0444 753111 - Fax 0444 753809 Mail protocollo@aulss8.veneto.it
PEC protocollo.centrale.aulss8@pecveneto.it
www.aulss8.veneto.it

Prot. n. 47057

Vicenza, 3.5.2021

Nucleo Ricerca Clinica AULSS 8 Berica

OGGETTO: Studio Clinico prot. PROVENT-C19 Registry. Titolo: Prone Positioning for invasively ventilated patients with COVID-19: an interactive, web-based, multicenter, observational registry.

Allo Sperimentatore Principale
Dott.ssa Silvia De Rosa
UOC di Anestesia e Rianimazione
Ospedale San Bortolo di Vicenza
e-mail: silvia.derosa@aulss8.veneto.it

Al Promotore
SIAARTI
Viale dell'Università, 11
00185 – Roma

E, p.c.

Al Direttore dell'UOC di Anestesia e Rianimazione
Ospedale San Bortolo di Vicenza
e-mail: segreteria.rianimazione@aulss8.veneto.it

Con Delibera n. 800 del 30.4.2021 il Direttore Generale dell'AULSS 8 Berica ha autorizzato la conduzione della sperimentazione specificata all'oggetto, valutata nella seduta del 13 aprile 2021 dal Comitato Etico per le Sperimentazioni Cliniche della Provincia di Vicenza, nominato con Deliberazione del Direttore Generale della ex ULSS n. 6 'Vicenza' n. 878 del 17 novembre 2016 e prorogato con successive Deliberazioni del Direttore Generale dell'ULSS n. 8 Berica n. 1912 del 11.12.2019 e n. 1745 del 18.11.2020.

Si ricorda che:

- lo Sperimentatore è tenuto a segnalare al Comitato Etico l'arruolamento del primo paziente;
- lo Sperimentatore è tenuto a trasmettere annualmente al Comitato Etico una comunicazione sullo stato di avanzamento dello studio;
- lo Sperimentatore è tenuto a trasmettere al Comitato Etico una notifica di conclusione dello studio e una relazione finale;
- il Comitato dovrà essere informato di eventuali reazioni avverse che dovessero verificarsi durante la conduzione della sperimentazione e di ogni successivo emendamento e modifica sostanziale del protocollo approvato;
- eventuali pubblicazioni di libri, articoli o relazioni tecnico-scientifiche in atti di convegni, con editori italiani o stranieri dovranno essere formalmente comunicate al CESC;
- nel caso di studio che preveda l'invio di farmaci alla UOC Farmacia Ospedaliera, si chiede al Promotore di inviare un'informazione preliminare alla UOC stessa relativamente al primo invio del farmaco sperimentale.

Cordiali saluti.

Nucleo Ricerca Clinica AULSS 8 Berica

All. scheda di valutazione allegata alla Delibera n.800 del 30.4.2021.

Responsabile del Procedimento: dott. Giuseppe Aprile
Referente procedura: Elda Dal Maso Segreteria Nucleo Ricerca Clinica
Tel. 0444 479660
e mail: elda.dalmaso@aulss8.veneto.it

COMITATO ETICO PER LE SPERIMENTAZIONI CLINICHE
DELLA PROVINCIA DI VICENZA

SEDUTA DEL 13 APRILE 2021

Componente	Figura professionale prevista dalla DGRV 1066/2013	Struttura di appartenenza	P= presente AG= assente giustificato A= assente
Dott. Giuseppe Aprile	Clinico	AULSS 8	P
Dott. Giuseppe Battaglia	Clinico	esterno	P
Sig.ra Marilena Bedin	Infermiere	esterno	P
Dott.ssa Eleonora Benetti	Pediatra	esterno	P
Dott.ssa Cristina Canova	Biostatistico	esterno	P
Dott. Francesco Caprioglio	Clinico	AULSS 8	P
Prof. Guido Francesco Fumagalli	Farmacologo	esterno	P
Dott.ssa Simonetta Morselli	Clinico	AULSS 7	P
Dott. Umberto Nardi	medico legale	AULSS 8	P
Dott. Paolo Pallini	Clinico	AULSS 8	P
Padre Stelio Pellegrinelli	esperto in bioetica	esterno	P
Ing. Alan Pettenà	esperto in dispositivi medici	esterno	P
Dott. Fabio Mario Randon	esperto in materia giuridica e assicurativa	AULSS 8	P
Dott. Marco Ruggeri	Clinico	AULSS 8	P
Dott. Michele Valente	medico di medicina generale territoriale	esterno	P
Dott.ssa Paola Valpondi	Farmacista del S.S.R.	AULSS 8	P
Dott. Narciso Zocca	rappresentante del volontariato	esterno	P
**Direttore Sanitario la cui partecipazione alle sedute del CE è prevista in caso di presentazione di progetti/studi della propria Azienda**			
Dott. Salvatore Barra			AG

Segreteria Scientifica: Dott.ssa Paola Valpondi

Scheda n. 2 facciata a) Sperimentazione n. 22/21

Protocollo: PROVENT-C19 Registry

Titolo: Prone Positioning for invasively ventilated patients with COVID-19: an interactive, web-based, multicenter, observational registry.

EudraCT n. ///

Promotore: Società Italiana Anestesia, Analgesia, Rianimazione e Terapia Intensiva - SIAARTI

CRO: ///

Scheda n. 2 facciata b) Sperimentazione n. 22/21

Unità Operativa: Anestesia e Rianimazione – Ospedale di Vicenza - AULSS n.8 Berica

Sperimentatore Principale: dott.ssa Silvia De Rosa

Relatore: dott.ssa Silvia De Rosa

Parere: Il CESC esprime **parere unico favorevole all'unanimità.**

Note:

Elenco documenti allegati alla domanda di autorizzazione:

- Lettera-intenti
- Protocollo PROVENT-C19 REGISTRY-Definitivo-17_03_21
- Sinossi PROVENT C19
- CRF
- Lettera informativa per il paziente
- Modulo di consenso informato
- Modulo di fattibilità locale
- Modulo_di_domanda_di_valutazione_e_dichiarazione_di_accettazione_dello_studio_clinico
- Dichiarazione sul conflitto interessi
- Dichiarazione sulla natura indipendente e no profit
- Dichiarazione sulla natura osservazionale
